# Coupling of Fibrin Reorganization and Fibronectin Patterning by Corneal Fibroblasts in Response to PDGF BB and TGFβ1

**DOI:** 10.3390/bioengineering7030089

**Published:** 2020-08-07

**Authors:** Miguel Miron-Mendoza, Dalia Vazquez, Nerea García-Rámila, Hikaru R. Ikebe, W. Matthew Petroll

**Affiliations:** Department of Ophthalmology, UT Southwestern Medical Center, Dallas, TX 75390, USA; miguel.miron@UTSouthwestern.edu (M.M.-M.); daliavazquez77@outlook.com (D.V.); nereagarciaramila@gmail.com (N.G.-R.); hikaru.ikebe@utsouthwestern.edu (H.R.I.)

**Keywords:** fibronectin, corneal fibroblasts, fibrin, 3-D matrices, collective cell migration

## Abstract

We previously reported that corneal fibroblasts within 3D fibrin matrices secrete, bind, and organize fibronectin into tracks that facilitate cell spreading and migration. Other cells use these fibronectin tracks as conduits, which leads to the development of an interconnected cell/fibronectin network. In this study, we investigate how cell-induced reorganization of fibrin correlates with fibronectin track formation in response to two growth factors present during wound healing: PDGF BB, which stimulates cell spreading and migration; and TGFβ1, which stimulates cellular contraction and myofibroblast transformation. Both PDGF BB and TGFβ1 stimulated global fibrin matrix contraction (*p* < 0.005); however, the cell and matrix patterning were different. We found that, during PDGF BB-induced cell spreading, fibronectin was organized simultaneously with the generation of tractional forces at the leading edge of pseudopodia. Over time this led to the formation of an interconnected network consisting of cells, fibronectin and compacted fibrin tracks. Following culture in TGFβ1, cells were less motile, produced significant local fibrin reorganization, and formed fewer cellular connections as compared to PDGF BB (*p* < 0.005). Although bands of compacted fibrin tracks developed in between neighboring cells, fibronectin labeling was not generally present along these tracks, and the correlation between fibrin and fibronectin labeling was significantly less than that observed in PDGF BB (*p* < 0.001). Taken together, our results show that cell-induced extracellular matrix (ECM) reorganization can occur independently from fibronectin patterning. Nonetheless, both events seem to be coordinated, as corneal fibroblasts in PDGF BB secrete and organize fibronectin as they preferentially spread along compacted fibrin tracks between cells, producing an interconnected network in which cells, fibronectin and compacted fibrin tracks are highly correlated. This mechanism of patterning could contribute to the formation of organized cellular networks that have been observed following corneal injury and refractive surgery.

## 1. Introduction

Following injury in most vascularized tissues, one of the first steps in wound healing is the formation of a provisional extracellular matrix (ECM), which is rich in fibrin. This matrix is essential to promote cell migration and invasion into the wounded area [1]. Fibrin fibers form a network that functions as a scaffold to provide the initial matrix for cells to attach, migrate, proliferate, and differentiate [2]. Fibronectin binds fibrin and contains binding sites for other ECM proteins, growth factors, and cells [3]. Most cells require fibronectin in order to bind to fibrin, and fibronectin is generally required for cells to migrate from the surrounding collagen matrix into the wound area [4,5]. Over time, cells degrade and replace this provisional ECM network with fibrotic tissue that is ultimately remodeled into a permanent ECM. In the case of wound healing in the cornea, which is avascular, the early stage of wound repair can also involve the deposition of a provisional matrix that functions as scaffold for cells to migrate [6], and fibrin and fibronectin are among the proteins present in provisional ECMs [7,8,9].

Following injury to the cornea, there are releases of prostaglandins, platelet-activating factor, growth factors and cytokines followed by apoptosis of the keratocytes adjacent to the wound [10]. Peptide growth factors such as IGF, PDGF BB, FGF, IL-1α and TGFβ1 [11,12,13,14] play an important role in modulating the keratocyte phenotype during in vivo corneal wound healing. Quiescent keratocytes residing in the well-organized collagen stroma adjacent to a wound proliferate and differentiate into migrating fibroblasts to repopulate the provisional ECM [15]. Fibroblastic cells invading the wound area transform into myofibroblasts that remove and synthesize new ECM, express alpha smooth muscle actin, and exert strong contractile forces [16,17,18]. The replacement of stromal keratocytes by fibrotic tissue produced by myofibroblasts alters corneal transparency, resulting in corneal haze and vision impairment [19,20]. TGFβ1 plays a central role in myofibroblast transformation of corneal keratocytes during wound healing [21,22,23]. In vivo studies of corneal injuries have shown an accumulation of fibronectin in the stroma during corneal fibrosis [21], and corneal myofibroblasts are known to express and organize fibronectin [24,25,26]. Remodeling of this fibrotic tissue is critical to restoring corneal transparency [27]; however, the mechanisms that direct the assembly and remodeling of the extracellular matrix following injury are not well understood. A better understanding of the processes governing the biomechanical interactions of corneal fibroblasts with their surrounding ECM could ultimately lead to more effective approaches to modulating the wound healing process in vivo. 

We previously demonstrated that ECM composition can modulate corneal behavior in 3-D culture in response to PDGF BB, which stimulates corneal fibroblast spreading and migration [28,29,30,31,32]. Specifically, whereas corneal fibroblasts generally move independently within 3-D collagen matrices, fibrin induces a switch to an interconnected, collective mode of cell spreading and migration that is independent of differences in ECM stiffness [33,34]. Furthermore, leading edge cells secrete and organize fibronectin into tracks during migration into 3-D fibrin matrices. These fibronectin tracks are used as conduits by the cells behind them, which leads to the development of an interconnected cell/fibronectin network [35]. Interestingly, networks of interconnected fibroblasts are also observed during in vivo corneal wound healing [7,36,37,38,39], and it is hypothesized that these networks may facilitate or enhance wound repopulation and contraction. However, the interrelationships between cell spreading, cell-induced ECM reorganization, and ECM deposition that may underlie network formation have not been determined. Furthermore, the effects of TGFβ1 on corneal keratocyte differentiation and mechanical behavior in 3-D fibrin matrices have not been assessed previously. In the present study, we use a fluorescent fibrin labeling technique and high resolution multi-dimensional imaging to explore the correlation between cell patterning, cell-induced fibrin ECM remodeling and fibronectin track formation by corneal fibroblasts in response to PDGF BB and TGFβ1.

## 2. Materials and Methods

### 2.1. Materials

Dulbecco’s modified Eagle medium (DMEM) and 0.25% trypsin/EDTA solution were purchased from invitrogen (Gaithersburg, MD, USA). Platelet-derived growth factor BB isotype (PDGF BB) was obtained from Upstate Biotechnology, Inc. (Lake Placid, NY, USA). Fetal bovine serum (FBS), fatty acid-free and fraction V bovine serum albumin (BSA), Hepes, Sodium bicarbonate, Thrombin from human plasma, and Anti-fibronectin antibody were obtained from Sigma-Aldrich (St. Louis, MO, USA). Penicillin-streptomycin-amphotericin B was obtained from lonza, Inc. (Walkersville, MD, USA). Fibrinogen was obtained from Enzyme Research Laboratories (South Bend, IN, USA). Alexa Fluor Phalloidin 546 was obtained from Molecular Probes, Inc. (Eugene, OR, USA). RNase (DNase free) was purchased from Roche (Indianapolis, IN, USA). For immunostaining, a rabbit anti-human fibronectin polyclonal antibody (sc-9068) and a mouse anti-human fibronectin monoclonal antibody were used (Santa Cruz Biotechnology, Inc., Santa Cruz, CA, USA). FITC conjugated goat anti-rabbit and goat anti-mouse secondary antibodies were obtained from Jackson ImmunoResearch (West Grove, PA, USA). For fibrin labeling, 5% Alexa fluor 405 NHS ester was obtained from Thermo fisher scientific (Waltham, MA, USA). FITC and Rhodamine fibronectin were obtained from Cytoskeleton Inc (Denver, CO, USA). GFP-Actin (rAV LifeAct, CMV, adenoviral vector) was purchased from ibidi USA (Madison, WI, USA).

### 2.2. Cell Culture

A previously published human corneal fibroblast cell line (HTK cells) [18] as well as primary cultures of rabbit corneal keratocytes (NRK cells) were used. HTK fibroblasts were maintained in tissue culture flasks with DMEM containing 10% FBS, supplemented with 1% penicillin, 1% streptomycin and 1% amphotericin B. NRK cells were isolated from the corneal stroma of rabbit eyes obtained from a slaughterhouse (Pel Freez, Rogers, AR, USA) as previously described [32,40]. NRK cell suspensions were seeded into tissue culture flasks and cultured in basal serum-free media for up to seven days prior to plating for experiments. Basal media consisted of DMEM containing pyruvate, HEPES, 1% RPMI vitamin mix, 1% 100× MEM non-essential amino acids, 100 μg/mL ascorbic acid and 1% penicillin/streptomycin/amphotericin B [40,41].

### 2.3. 3D Matrix Model

Cell spreading and patterning were assessed in fibrillar fibrin matrices. In this model, 150 µL of fibrin (1 mg/mL) containing 5 × 10^5^ cells was poured onto glass bottom dishes (MatTek, model P35GC-1.5-14-C, Ashland, MA, USA). Fibrinogen was warmed for 20 min and mixed with DMEM to achieve a final concentration of 1 mg/mL, and the solution was mixed with 0.5 U/mL thrombin to initiate polymerization. To label fibrin fibers, 5% Alexa Fluor™ 405 NHS Ester (Succinimidyl Ester) was mixed with fibrinogen before warming [42]. Samples were then placed for 30 min in a humidified incubator (37 °C, 5% CO_2_) to polymerize. Matrices were then gently rinsed twice with DMEM to remove any excess thrombin and NHS ester. Next, 2 mL of serum-free basal media, or basal media supplemented with either PDGF BB (50 ng/mL) or TGFβ1 (5 ng/mL) was added to the dishes and samples were cultured for 2–4 days.

### 2.4. Fluorescent Labeling and Static Imaging of 3-D Constructs

For Fluorescent labeling, constructs were fixed with 3% paraformadehyde in phosphate buffer, PBS, for 10 min, and permeabilized with 0.5% Triton X-100 in PBS for 15 min. Samples were blocked with 1% BSA fraction V in PBS for 1 h. Samples were then washed three times, 20 min per wash. For fibronectin labeling of HTK cells, samples were incubated for 120 min with a rabbit anti-human fibronectin polyclonal antibody at a ratio of 1:100, washed for 60 min with PBS, and then incubated for 60 min with FITC conjugated goat anti-rabbit secondary antibody. For fibronectin labeling of NRK cells, samples were incubated for 120 min with a mouse anti-human fibronectin monoclonal antibody at a ratio of 1:100, washed for 60 min with PBS, and then incubated for 60 min with FITC conjugated goat anti-mouse secondary antibody. For α-SM labeling of both HTK and NRK cells, samples were blocked with 1% BSA fraction V in PBS for 1 h following permeabilization. Samples were then washed three times, 20 min per wash, and then incubated with primary mouse anti-human α-SMA antibody (1:600, Sigma, St. Louis, MO, USA) for 2 h at 37 °C. Cells were then washed three times, 20 min per wash, and then incubated with FITC conjugated goat anti-mouse secondary antibody (1:200). After immunolabeling, samples were washed for 30 min, incubated with Alexa Fluor 546 Phalloidin (1:150 ratio) for 60 min and then washed for an additional 30 min. All staining procedures were carried out in the original culture plates to avoid cell or matrix distortion.

Fluorescence Images were collected with a laser confocal microscope (Leica SP8, Heidelberg, Germany). A UV laser (405 nm) was used to image fibrin, an Argon laser (488 nm) was used for fibronectin and a GreNe (543 nm) laser was used for F-actin. Images were acquired sequentially to avoid cross talk between the channels. A stack of optical sections was acquired by changing the position of the focal plane in the z-direction with a step size of 1 or 2 µm, using a 63× oil immersion objective or 25× water immersion objective, respectively.

### 2.5. Live Cell Imaging

For dynamic imaging of cytoskeletal dynamics, cell-induced fibrin reorganization and fibronectin patterning, cells were prepared for live-cell imaging as follows. For visualization of F-actin, cells were incubated for 48 h with the LifeAct adenoviral vector (1 μL/mL). LifeAct is a 17-amino acid peptide that stains filamentous actin (F-actin) structures in living or fixed eukaryotic cells and tissues. It has a TagGFP2 marker to allow visualization of the labeled F-actin using FITC optics. For visualization of fibronectin, 5 mg/mL Rhodamine fibronectin (rhodamine molecules covalently linked to pure fibronectin) was added to the media 60 min prior to the start of time-lapse imaging. For imaging of these fluorescently labeled samples, a Leica SP8 confocal microscope with an environmental chamber (Life Imaging Services, Basel, Switzerland) was used. A UV laser (405 nm) was used to image fibrin, an argon laser (488 nm) was used for imaging F-actin and a GreNe (543 nm) laser was used for Rhodamine fibronectin. 3-D stacks of 10–15 images (1–2 micron steps) of labeled fibrin, fibronectin and F-actin were taken at 20 min intervals using a 63× oil immersion objective. Live cell imaging was carried out beginning 6–24 h after cell plating depending on the experimental condition.

### 2.6. Global Matrix Contraction

To calculate the percentage of cell-induced fibrin matrix contraction, immediately after media was added, the height of the fibrin matrix was measured and recorded. After 48 h of incubation, the height of the fibrin matrix was measured again and recorded. To measure matrix height, we used a calibrated phase contrast inverted microscope with a 10× objective (Nikon Eclipse Ti; Nikon, Tokyo, Japan). Measurements were made on at least four matrices for each experimental condition.

### 2.7. Image Processing, 3D Reconstruction, Cell Connectivity and Morphometric Analysis

For images collected from fixed and labeled samples, image processing and 3D reconstruction of z-stacks were carried out using MetaMorph software (version 7.7, Molecular Devices, Sunnyvale, CA, USA). Maximum intensity projection images were generated from the image stack from each channel, and combined using the color overlay function. The 3D reconstruction function was then applied in some cases to allow visualization of cell-ECM interactions over a range of projection angles. For time-lapse experiments, maximum intensity projection images were generated from a subset of planes within the z-stack collected at each time point, and combined to from a single time-lapse movie.

To calculate the percentage of cells that were in contact with other cells, the 3D reconstructed sample was viewed while rotating over 360 degrees. If cell extensions from two different cells were overlapping (touching) for the entire 360-degree rotation, this counted as a positive connection. The percentage of cells with at least one connection with another cell was then calculated.

Cell morphology measurements were made using MetaMorph. Projected cell length, breadth and area were calculated by outlining the maximum intensity projection image of a cell (generated from the f-actin z series), thresholding, and applying the Integrated Morphometry Analysis (IMA) routine. The length is calculated by IMA as the longest cord between any two points in the cell, and the breadth is the width perpendicular to that cord.

### 2.8. Correlation between F-Actin, Fibrin and Fibronectin Patterning

To quantify the relationships between F-actin, fibronectin, and fibrin patterning, images were collected from samples that were fixed and labeled as described above. Using a 25× water immersion objective, stacks of optical sections were acquired by changing the position of the focal plane in the z-direction with a step size of 2 µm. Maximum intensity projection images were generated from the image stack from each channel. Using Image J software, lines were traced across the fibrin tracks between neighboring cells and across the cell body, using the same *x*-*y* coordinates for each channel (Supplementary Figure S3). Subsequently the pixel intensity values along the traced lines for each channel were loaded into an Excel file. Using the Correlation function in Excel, the pixel intensity values from each channel were then compared (Actin-fibronectin, Actin-Fibrin, fibronectin-fibrin) and the corresponding correlation coefficient (R) calculated.

### 2.9. Statistical Analysis

Statistical analyses were performed using SigmaPlot (version 12.5, Systat Software, Inc., San Jose, CA, USA). *p*-values for proportions were calculated using chi-square analysis. For comparison of mean values of two samples, a two tailed *t*-test was used. For comparisons between more than two sample means ANOVA was used for paramtetric data and ANOVA on ranks was used for non-parametric data.

## 3. Results

### 3.1. Cell-Induced Matrix Contraction in Response to PDGF BB and TGFβ1

**Figure 1:**
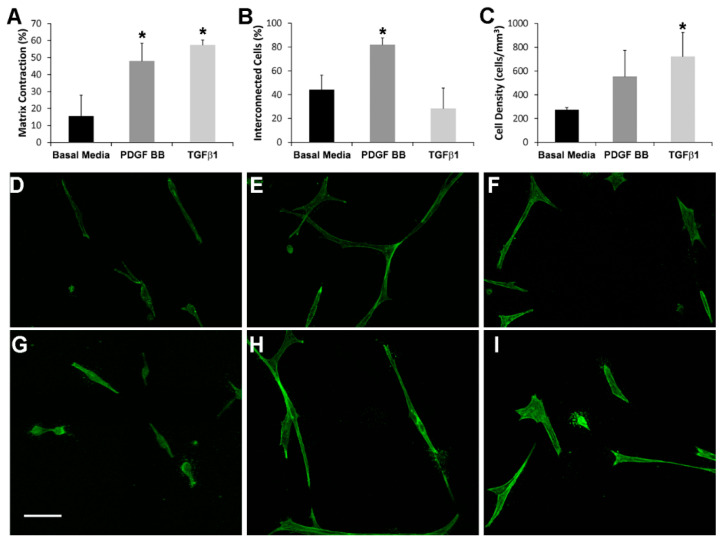
(**A**) Percentage of cell-induced fibrin matrix contraction after 48 h of incubation. Both PDGF BB and TGFβ1 stimulate significant global matrix contraction (as indicated by a decrease in matrix height) as compared to basal media (* *p* < 0.005, ANOVA with Holm-Sidak multiple comparison procedure). Data are the mean ± SD of the % decrease in height from four experiments. (**B**) The percentage of connections between neighboring cells is significantly higher in PDGF BB as compared to basal media and TGFβ1 (* *p* < 0.005, ANOVA with Holm-Sidak multiple comparison procedure). The percentage of interconnected cells (cells connected to at least one neighboring cell) was quantified from maximum intensity projections of 100 μm thick z-scans viewed over a 360-degree range of projection angles. The results are the mean ± SD from four separate matrices for each condition. (**C**) The cell density after 48 h of culture is significantly higher in TGFβ1 as compared to basal media (* *p* < 0.05, ANOVA with Holm-Sidak multiple comparison procedure). Cell densities were measured by counting nuclei in 100 μm thick z-scans. The results are the mean ± SD from four separate matrices for each condition. (**D**–**I**) Representative maximum intensity projections of F-actin organization in HTK cells cultured for 48 h in 3-D fibrin matrices showing differences in cell connectivity and morphology following culture in basal media (**D**,**G**), PDGF BB (**E**,**H**) and TGFβ1 (**F**,**I**). Note that cells are more elongated and more interconnected in PDGF BB as compared to basal media or TGFβ1. Scale bar = 75 μm.

In order to compare global cell-induced matrix contraction in response to PDGF BB and TGFβ1, we measured the decrease in matrix height after 48 h of incubation. Figure 1A shows the percentage of 3D fibrin matrix contraction for PDGF BB, TGFβ1, and no growth factor (basal media). The amount of fibrin matrix contraction was similar between PDGF BB or TGFβ1, and was significantly greater than that produced by basal media (* *p* < 0.005). However, the pattern of cell-matrix interactions was different. As shown in Supplementary Video S1, cells in PDGF BB actively extended and retracted pseudopodial processes during the first 24 h after plating, and this produced transient tractional forces on the fibrin ECM at the leading edge of pseudopodia. In contrast, cells in TGFβ1 were much less motile (Supplementary Video S2) and appeared to exert contractile forces across the entire cell body (from end to end). Over time, cells in PDGF BB continued to elongate and interconnect with neighboring cells (Figure 1E,H), whereas cells in basal media (Figure 1D,G) and TGFβ1 (Figure 1F,I) were more compact and isolated (see also Figure S1). Quantitative analysis demonstrated that the percentage of connections between neighboring cells was significantly higher in PDGF BB as compared to basal media and TGFβ1 (Figure 1B, *p* < 0.005). As shown in Figure 1C, this increase in connectivity was not due to a higher cell density in PDGF BB as compared to TGFβ1. In fact, cell density at 48 h was higher for both TGFβ1 and PDGF BB as compared to basal media, most likely due in part to increased matrix compaction in these conditions. This difference reached statistical significance for TGFβ1 (*p* < 0.05). Similar results were obtained using NRK cells (not shown).

Quantitative assessment of cell morphology was carried out to evaluate the apparent differences in cell spreading between culture conditions. As shown in Table 1, cell area, cell length and the length/breadth ratio were all significantly higher in HTK cells cultured in PDGF BB as compared to basal media or TGFβ1. This is consistent with our qualitative observations that PDGF BB stimulates cell spreading and elongation of corneal fibroblasts. Similar results were obtained for NRK cells (Supplementary Table S1).

### 3.2. Fibrin Reorganization and Fibronectin Patterning during PDGF BB Induced Cell Spreading

We previously reported that cells interacting with fibrin matrices form fibronectin “tracks” [35]. These fibronectin secretions are required for cell attachment to the fibrin ECM and are organized by cells during migration. Fibronectin tracks subsequently promote the development of interconnected streams of cells that result in collective cell migration. In order to determine whether cell-induced fibrin fiber reorganization plays a role in mediating fibronectin track formation and/or cell patterning, we applied a technique for fluorescently labeling fibrin fibers in our 3D matrices [42]. The Alexa Fluor™ 405 NHS Ester is a dye that labels the primary amines of proteins and can therefore conjugate to virtually any protein. We used the NHS 405 to label the fibrinogen used for our 3-D fibrin matrices. The blue emission spectrum of the NHS 405 allowed us to preserve the red and green spectrum for imaging other cell/ECM proteins of interest such as fibronectin and F-actin. To experimentally verify that the fluorescent signal from the NH3 ester dye was specific to fibrin labeling, we compared the labeling patterns of fibrin, fibronectin and F-actin at high magnification in several samples. As shown in Supplementary Figure S2, there is no indication that there is any residual NH3 ester dye that is labeling the cell secreted fibronectin or binding to the cell membrane.

**Figure 2:**
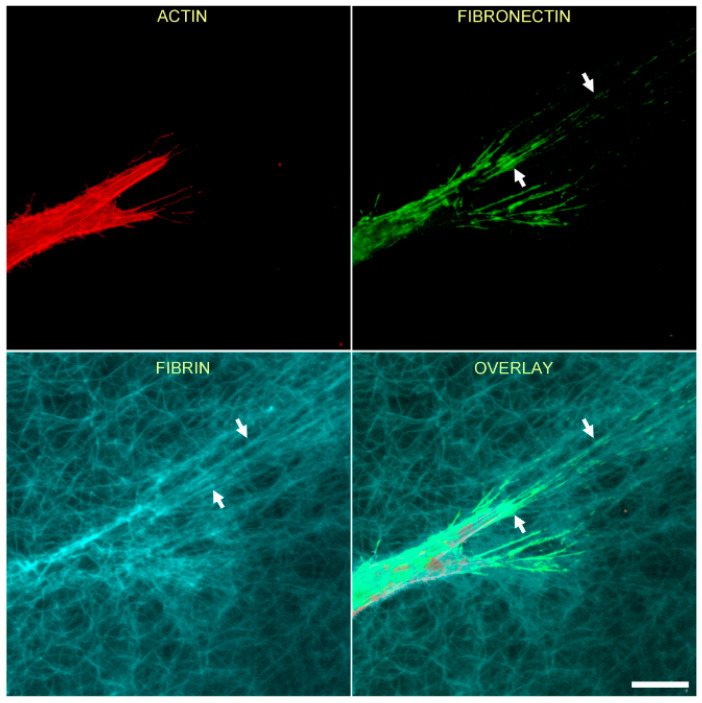
Corneal fibroblasts organize fibronectin along compacted lines of fibrin fibers. HTK cells were cultured in a fluorescently labeled 3D fibrin matrix for 48 h in media containing PDGF BB (to stimulate cell spreading). Subsequently, cells were fixed and labeled for F-actin and fibronectin. Images are maximum intensity projections of z-series from each individual channel. Fibrin is compacted and aligned in front of the pseudopodial extensions, presumably due to tractional force generation. Fibronectin is organized into lines that colocalize with the compacted fibers (arrows). Scale bar is 20 µm.

Figure 2 shows the relationship between F-actin organization and fibrin and fibronectin patterning for an isolated HTK cell following cell spreading. In this experiment, cells were cultured for 48 h in media supplemented with PDGF BB, then fixed and labeled with phalloidin (for F-actin) and an anti-fibronectin antibody. Note that at the leading edge of the cell, fibrin fibers have been pulled into alignment with the pseudopodial process, and cell-secreted fibronectin is colocalized with these fibrin fibers (arrows).

**Figure 3:**
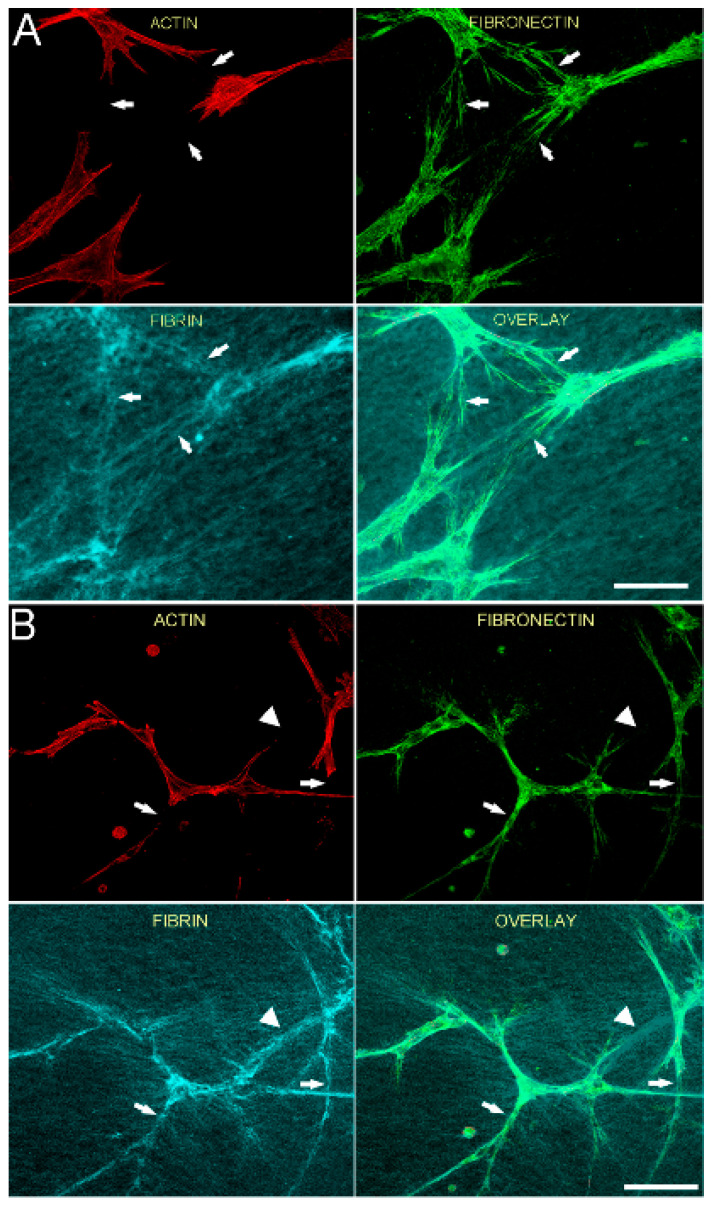
Fibronectin tracks colocalize with compacted lines of fibrin fibers between cells. HTK cells (**A**) or NRK cells (**B**) were cultured in a fluorescently labeled 3D fibrin matrix for 48 h in media containing PDGF BB (to stimulate cell spreading). Subsequently, cells were fixed and labeled for F-actin and fibronectin. Images are maximum intensity projections of z-series from each individual channel. For both HTK and NRK cells, a network of fibronectin tracks is observed along and between groups of migrating cells. When present, fibronectin tracks colocalize with lines of fibrin compaction (arrows). In one area, fibrin matrix compaction is observed without corresponding fibronectin labeling (B, arrowhead). Scale bars are 150 µm.

We previously showed that tracks of fibronectin are found between neighboring corneal fibroblasts cultured in PDGF BB, resulting in an interconnected cell/fibronectin network [35]. To assess cell-cell interactions and fibrin/fibronectin network formation, we used a lower magnification objective to collect images showing groups of neighboring cells. Figure 3 is a lower magnification image showing the relationship between F-actin, fibronectin and fibrin fibers for both HTK (A) and NRK (B) cells, following incubation for 48 h in media containing PDGF BB. Fibronectin is observed both around and between cells. In addition, fibrin fiber reorganization by cells results in the formation of an interconnected network of compacted lines of fibrin fibers. Importantly, fibronectin tracks co-localize with these compacted lines of fibrin (arrows). However, fibrin matrix compaction was sometimes observed without corresponding fibronectin labeling (B, arrowhead).

These findings suggest that cells activated with PDGF BB pull and organize fibrin fibers as they spread and interconnect with one another, while simultaneously secreting and organizing fibronectin. Once cells move away, these fibrin/fibronectin tracks remain. To assess the dynamic interactions which lead to this organization, time-lapse imaging was used. As shown in Supplementary Video S3, during cell spreading in media containing PDGF BB, fibronectin (red in overlay on left panel, also shown alone in right panel) is deposited and organized simultaneously with the generation of tractional forces at the leading edge of pseudopodia (arrows). This is observed in cells with and without LifeAct expression (green in left panel). Note that in one area a cell lays down fibronectin during spreading, and the fibronectin remains after the processes retract (circle). In addition, cell spreading and fibronectin deposition is observed along compacted fibrin tracks (cyan in left panel) between cells (arrow head).

### 3.3. Fibrin Reorganization and Fibronectin Patterning during TGFβ1 Induced Cell Contraction

**Figure 4:**
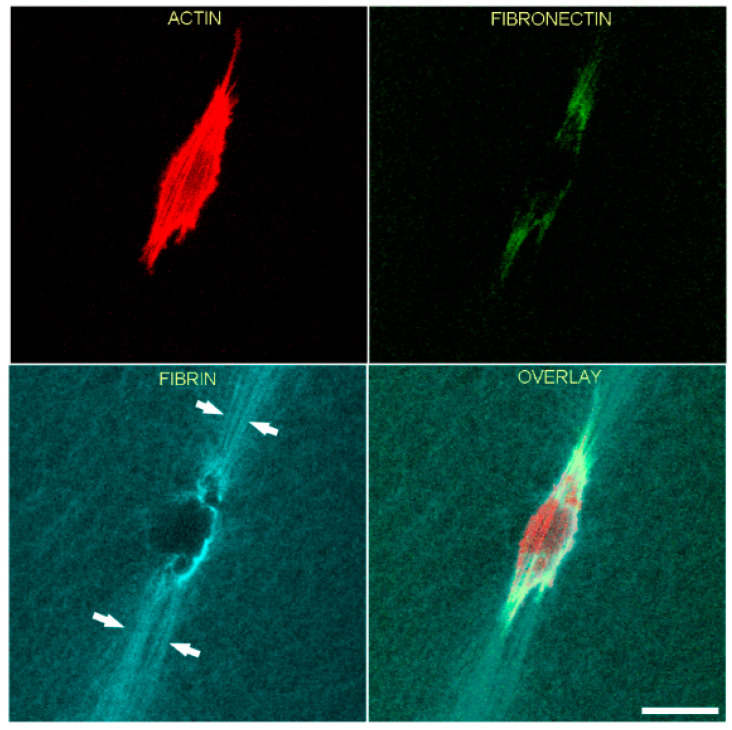
Representative image of a living HTK cell showing fibronectin and fibrin organization in a 3-dimensional fibrin matrix. LifeAct-transfected cells were cultured overnight with media containing TGFβ1 to stimulate cell contractility. One hour before cells were placed in microscope for recording, fluorescent fibronectin was added to the media. Maximum intensity projections of z-series collected one hour after the start of the time-lapse are shown. Significant fibrin fiber compaction and alignment are present at both the front and rear of the cell (arrows). Compacted fibrin is coaligned with the intracellular F-actin stress fibers. Fibronectin labeling is limited to the cell area. Scale bar is 40 µm.

In order to gain more insights into the relationships between cell spreading, ECM patterning and fibronectin network formation, we used TGFβ1, which is known to activate the contractile machinery of corneal keratocytes without inducing significant cell spreading and migration [43,44]. As shown in Figure 4, significant compaction and alignment of fibrin is present at both the front and rear of HTK cells cultured in TGFβ1 (arrows). In contrast, fibronectin labeling is limited to the cell area. Compacted fibrin was co-aligned with prominent intracellular F-actin stress fibers.

**Figure 5:**
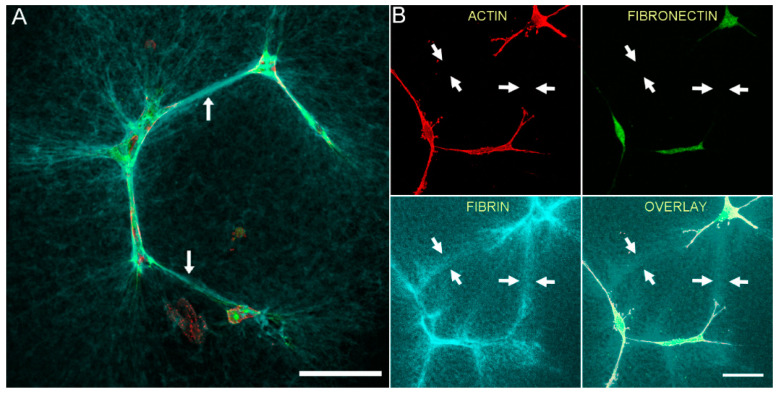
Neighboring contractile cells create an interconnected network of compacted fibrin fibers, without the formation of a fibronectin network. HTK cells (**A**) or NRK cells (**B**) were cultured in a fluorescently labeled 3D fibrin matrix (cyan) for 48 h in media containing TGFβ1 (to stimulate cell contraction). Subsequently, cells were fixed and labeled for F-actin (red) and fibronectin (green). Images are maximum intensity projections of z-series from each individual channel. For both HTK and NRK cells, compacted lines of fibrin fibers developed between adjacent cells (arrows), forming an interconnected network. In contrast, fibronectin labeling was only observed where cells were present. Scale bars are 75 µm.

To assess cell-cell interactions and fibrin/fibronectin network formation by cells cultured in TGFβ1, we used a lower magnification objective to collect images showing groups of neighboring cells. Figure 5 shows HTK (A) and NRK (B) cells fixed after 48 h of incubation with TGFβ1 and labeling for F-actin and fibronectin. Neighboring cells created a pronounced interconnected network of compacted fibrin fibers (cyan). Although fibronectin labeling was co-localized with the cells, it was often absent from compacted tracks of fibrin between cells (arrows in Figure 5, see also Supplementary Video 4). These observations demonstrate that in contrast to cell spreading in PDGF BB, TGFβ1-induced cell contraction results in compaction of fibrin between cells without corresponding fibronectin track formation.

### 3.4. TGFβ1 Induced Myofibroblast Transformation

**Figure 6:**
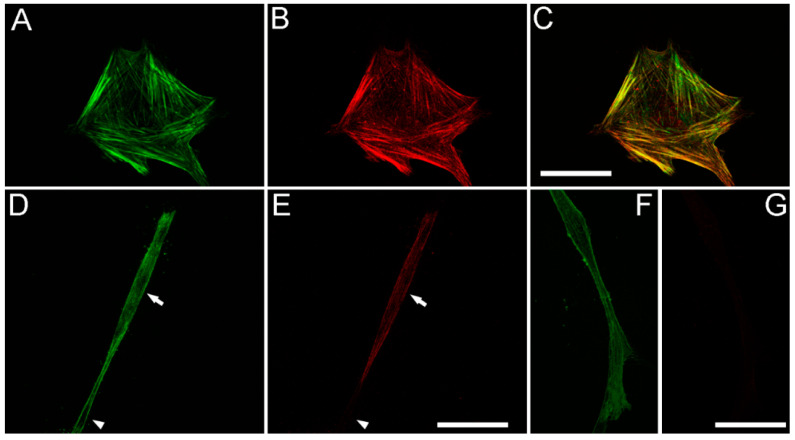
TGFβ1 induces α-SM actin expression in HTK cells. (**A**,**D**,**F**) show F-actin labeling. (**B**,**E**,**G**) show α-SM actin labeling. (**A**–**C**) HTK cells cultured in TGFβ1 that were on the bottom of the matrix and interacting with the rigid glass substrate had strong α-SM actin labeling, which was colocalized with F-actin labeled stress fibers (**C**, color overlay). Note that these cells also had a broad morphology. (**D**,**E**) Most of the HTK cells inside the fibrin matrix also had positive labeling for α-SM actin, however the labeling intensity was weaker. Cells inside the fibrin ECM maintained a more bipolar morphology even when labeling positive for α-SM actin. Both α-SM actin positive (arrows) and negative (arrowhead) cells were observed. (**F**,**G**) HTK cells cultured in PDGF BB were negative for α-SM actin, as indicated by weak background labeling. Scale bars are 50 µm.

TGFβ1 has been shown to play a central role in stimulating myofibroblast transformation of corneal keratocytes, as indicated by expression of stress fibers containing α-smooth muscle actin (α-SMA) [45], and production of abnormal, fibrotic ECM [46,47,48]. However, myofibroblast transformation can be impacted by substrate stiffness and composition, and the effect of TGFβ1 on corneal keratocytes inside fibrin matrices has not been evaluated. To assess whether TGFβ1 induced myofibroblast transformation of HTK cells in our 3-D fibrin matrix model, we used labeling of α-SM actin. As shown in Figure 6A–C, HTK cells cultured in TGFβ1 that were on the bottom of the matrix and interacting with the rigid glass substrate had strong α-SM actin labeling. The α-SM actin was colocalized with F-actin labeled stress fibers. Note that these cells also had a broad morphology which is typical for corneal myofibroblasts. Most of the HTK cells inside the fibrin matrix also had positive labeling for α-SM actin (Figure 6D,E); however, the labeling intensity was weaker. Interestingly, cells inside the fibrin ECM maintained a more bipolar morphology even when labeling positive for α-SM actin. Both positive (arrows) and negative (arrowhead) cells were observed. HTK cells cultured in serum free media or PDGF BB (Figure 6F,G) were negative for α-SM actin both on the bottom of the ECM and inside the fibrin, as indicated by weak background labeling. NRK cells were negative for α-SM actin at 48 h for all conditions studied (not shown), consistent with previous studies suggesting that full transformation from a quiescent keratocyte to a myofibroblast in 3-D culture takes 4–5 days.

### 3.5. Correlation of Fibronectin Tracks and Compacted Fibrin Fibers in PDGF BB and TGFβ1 Cultured Cells

**Figure 7:**
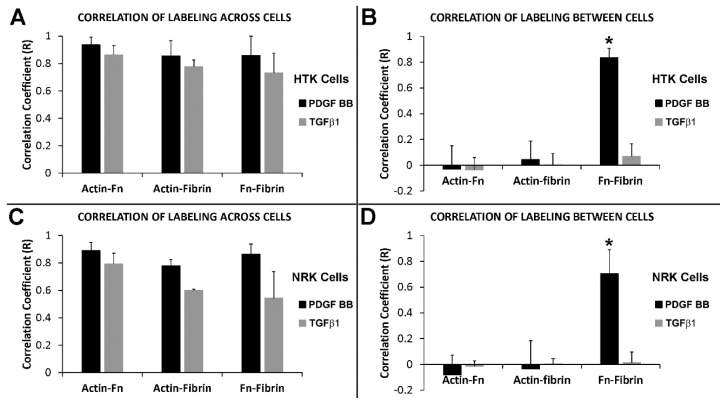
Correlation between actin, fibrin and fibronectin patterning. HTK cells (**A**,**B**) or NRK cells (**C**,**D**) were cultured in a fluorescently labeled 3D fibrin matrix for 48 h in media containing PDGF BB (to stimulate cell spreading) or TGFβ1 (to induce cell contractility). Subsequently, cells were fixed and labeled for F-actin and fibronectin. Correlation coefficients were calculated by comparing the relationship between actin, fibronectin, and fibrin fibers across the cell body and between neighboring cells. Across the cell body (**A**,**C**), high correlations were found for both PDGF BB and TGFβ1. Between neighboring cells (**B**,**D**), the only significant correlation found was between fibrin and fibronectin when cultured with PDGF BB (* three way ANOVA, *p* < 0.001). Means and standard deviations are calculated from three independent experiments (each with 5–10 neighboring cell pairs per culture condition).

The results with cells cultured with PDGF BB show that tracks of fibronectin are found between neighboring cells along with the formation of an interconnected network of compacted lines of fibrin fibers. On the other hand, while our results with contractile cells in TGFβ1 show similar formation of an interconnected network of compacted lines of fibrin fibers, tracks of fibronectin are generally absent between cells. To quantify these differences in patterning, we calculated the correlation between actin, fibrin and fibronectin labeling in both PDGF BB and TGFβ1 (see Supplementary Figure S3 for example). Figure 7 shows the correlation coefficient graphs both across the cell body and between cells following culture in PDGF BB and TGFβ1 for both HTK cells (Figure 7A,B) and NRK cells (Figure 7C,D). The results show that across cell body, the actin cytoskeleton, fibronectin, and fibrin reorganization are highly correlated for both PDGF BB and TGFβ1. On other hand however, these results also show that between neighboring cells, the only significant correlation was between fibrin and fibronectin in PDGF BB culture media. These findings are consistent with the premise that cells activated with PDGF BB compact fibrin fibers as they spread and interconnect with one another, while simultaneously secreting and organizing fibronectin. Once cells move away, these fibrin/fibronectin tracks remain. In TGFβ1 cells are more stationary and have fewer of these transient interactions with other cells, thus fibronectin tracks are rarely left behind. It should be noted that when present, fibronectin tracks were always colocalized with fibrin compaction.

## 4. Discussion

Cell-ECM mechanical interactions mediate numerous biological processes including developmental morphogenesis, wound healing and cancer metastasis. In addition to biochemical regulation of cell differentiation, dynamic feedback between cellular force generation, protein expression and patterning, and ECM mechanical, biochemical and biophysical properties plays a critical role in modulating cell motility and mechanical behavior [42,49,50,51,52]. In this study, we used high resolution multi-dimensional imaging to explore the correlation between cell-induced fibrin ECM remodeling and fibronectin track formation during corneal fibroblast spreading and contraction in a 3D culture model. In previous publications, we used confocal reflection imaging to visualize collagen fibril organization in 3D matrices [53]. However, it is generally difficult to obtain confocal reflection images of fibrin fibrils within 3D fibrin matrices, presumably due to increased reflectivity and less ECM porosity. In addition, the signal from confocal reflection imaging is dependent on the angle of the fibril, and can include contributions from other ECM proteins such as fibronectin. By directly labeling fibrin fibers with a fluorescent tag, we were able to directly investigate how cell-induced compaction correlates with fibronectin (Fn) secretion and track formation in response to two growth factors present during corneal wound healing: PDGF BB and TGFβ1.

PDGF BB has been shown previously to stimulate Rac-induced spreading of dermal and corneal fibroblasts in 3-D collagen matrices, along with significant tractional force generation by extending pseudopodial processes [31,54]. In the current study, we demonstrate for the first time that PDGF BB also stimulates fibroblast elongation and spreading in fibrin matrices, and this process induces significant local matrix reorganization. The process of cell spreading in media containing PDGF BB showed cells simultaneously secreting and organizing Fn while compacting fibrin fibers at the leading edge. Fn matrix assembly requires the polymerization of multiple molecules together into fibrils [55]. The self-assembly of Fn fibrils is a cell mediated process that involves Fn recognition by cell surface receptors such as integrins, and the elongation of its structure to expose cyptic binding sites necessary for fibrillogenesis [3,56,57]. The Fn cryptic binding sites can be exposed by tension, heat denaturation, or cleavage of specific domains (e.g., FnIII) [58,59]. Fn is therefore mechanosensitive and the application of force can induce conformational changes in the Fn molecule [60]. For instance, the Fn type III repeat contains hydrogen bonds which are thought to be susceptible to unfolding by pressure or mechanical tension. In our experiments, the bundles of compacted lines of fibrin fibers observed in fibrin ECM are the result of mechanical forces applied by cells. Specifically, in order to spread or migrate, cells grab and pull individual fibrin fibers to exert tractional forces to extend protrusions and move forward. The forces generated could have the potential to expose Fn cryptic binding sites that result in Fn self- assembly; which is observed in our experiments as Fn track formation. 

Our studies also show that after cells retract their processes, Fn tracks remain in the fibrin ECM. Many cells require Fn to attach to fibrinogen and fibrin, and Fn is required for dermal fibroblast invasion of fibrin matrices [5,61]. In previous studies, we have shown that Fn mediates attachment of corneal fibroblasts to fibrin via α5β1 integrin, and blocking Fn binding inhibits cell spreading and migration in fibrin matrices [35]. The α5β1 integrin is the primary receptor for binding to soluble Fn and requires the combination of the RGD sequence and the synergy site in Fn, resulting in specificity of Fn binding [3,62]. Integrins promote Fn-Fn interactions outside the cells that can result in Fn fibril formation [56]. Initial Fn binding to α5β1 integrin results in receptor clustering which brings together Fn dimers [63]. Cell tractional forces or Fn-Fn interactions can both cause conformational changes to the α5β1-Fn bond and extend the cluster to induce Fn fibril formation [64]. In our case, the initial attachment mediated by α5β1 integrin seems to be the first step required for Fn fibril assembly. Continued accumulation of Fn dimers likely serve to lengthen and thicken Fn fibrils. With time Fn fibrils can be further stabilized inside fibrillar networks, producing a deoxycholate-insoluble Fn matrix [55,65]. It has not been determined whether the Fn fibril network is deoxycholate insoluble in our model. 

Using a nested cell migration model, we previously demonstrated that fibronectin tracks left behind by leading edge cells serve as paths for other cells and can function as a guide for cell migration, similar to those reported during tissue development [66,67]. In the current study, we found that a corresponding network of compacted fibrin tracks is produced during cell spreading and migration in PDGF BB. Time-lapse imaging suggests that cells exert tractional forces that compact and align fibrin fibrils in front of advancing pseudopodial extensions, while simultaneously secreting and organizing fibronectin. This pattern of fibrin matrix remodeling is similar to tractional structuring that occurs in collagen matrices [68,69], a process that is believed to be operative during wound healing [70]. The compacted lines of fibers likely increase the ECM stiffness and provide more mechanical resistance than surrounding individual fibers. Fibroblasts migrate toward stiffer regions in pre-fabricated 3D matrices with directional gradients in collagen density [71]. A similar result has been observed with polyacrylamide substrates of different stiffness, where cells migrate preferentially toward stiffer substrates, a phenomenon called durotaxis [72]. Taken together, these results suggest that cells create lines of tensioned fibrin fibers by exerting tractional force, and cells preferentially spread and migrate along these tracks. We demonstrate for the first time that during this dynamic process, cells also secrete and organize fibronectin networks.

In order to gain more insights into the relationships between cell spreading, ECM remodeling and fibronectin network formation, we used TGFβ1. TGFβ1 plays a central role in myofibroblast transformation of corneal keratocytes during wound healing, and has been shown to stimulate corneal keratocyte contractility and local ECM reorganization in 3-D collagen matrices. In this study, we demonstrate for the first time that TGFβ1 also stimulates cell-induced ECM compaction and reorganization in 3-D fibrin matrices. We also assessed the expression of α-SM actin, an indicator of myofibroblast transformation. Interestingly, HTK cells cultured in TGFβ1 that were on the bottom of the matrix and interacting with the rigid glass substrate had strong α-SM actin labeling, which was colocalized with F-actin labeled stress fibers. These cells also had a broad morphology which is typical for corneal myofibroblasts. Most of the HTK cells inside the fibrin matrix also had positive labeling for α-SM actin; however, the labeling intensity was weaker and cells inside the fibrin ECM maintained a more bipolar morphology even when labeling positive for α-SM actin. This suggests only partial transformation to a myofibroblast phenotype at the 48 h time point analyzed. These results are consistent with previous studies using 3-D collagen matrices which showed more rapid myofibroblast transformation in response to more rigid ECM [32], and highlight the differences in cell behavior between rigid 2-D substrates and fibrillar 3-D matrices. Note that long term culture were avoided because of the impact that cell proliferation could have on our results.

Significant compaction and alignment of fibrin was observed at both the front and rear of corneal fibroblasts cultured in TGFβ1. However, fibronectin labeling was generally limited to the cell area. In regions of higher cell density, although corneal fibroblasts cultured in TGFβ1 were able to pull fibers inward to create compacted lines of fibrin between cells, they were less motile and did not spread or secrete fibronectin along these compacted fibers. These results further demonstrate that the compaction of fibrin fibers is produced by tractional forces exerted by the cells, whereas fibronectin is only secreted and organized in areas where cells have spread and attached to the fibrin ECM. Following incisional surgery, corneal fibroblasts form an interconnected network as they migrate into the wound space, and these interconnections are hypothesized to facilitate wound contraction [7,36]. Furthermore, following a transcorneal freeze injury or keratectomy surgery in the rabbit, highly aligned streams of interconnected fibroblasts migrate into the injured stromal tissue [37,38]. Further studies are needed to determine whether networks of ECM remodeling and protein secretion similar to those observed in the current study also mediate these cell patterning behaviors in vivo.

## 5. Conclusions

Networks of interconnected fibroblasts are often observed during in vivo corneal wound healing, and it is hypothesized that these networks may mediate wound repopulation and closure. We previously demonstrated that corneal fibroblasts form interconnected cell-fibronectin networks when cultured in 3-D fibrin matrices. However, the role of local cell-induced matrix reorganization in mediating this process has not been studied. Furthermore, previous work focused on cell migration induced by PDGF BB, and the effects of TGFβ1 (a key wound healing cytokine) on corneal fibroblast behavior in 3-D fibrin matrices has not been assessed. In this study, we show for the first time that both PDGF BB and TGFβ1 induce significantly more cell-induced fibrin matrix reorganization as compared to basal media; however, the pattern of cell spreading is different, and cells in PDGF BB are more elongated and interconnected as compared to cells in either basal media or TGFβ1. Quantitative analysis demonstrated that local cell-induced fibrin reorganization sometimes occurred independently of fibronectin deposition, particularly during TGFβ1 induced cell contraction. However, fibronectin deposition was associated with local cell-induced matrix reorganization when interconnected cell networks formed in PDGF BB. We also demonstrate that TGFβ1 can induce myofibroblast transformation of corneal fibroblasts in 3-D fibrin matrices, and that the degree of transformation is likely dependent on the local stiffness of the ECM. Overall, our results show for the first time that fibrin reorganization and fibronectin patterning can occur independently. Nonetheless, when cells are spreading or migrating, both events seem to be coordinated, as cells secrete and organize fibronectin as they preferentially spread along compacted fibrin tracks around and between cells.

## Figures and Tables

**Table 1:** HTK Cell Morphology.

	Basal Media	PDGF BB	TGFβ1	Basal Media vs. PDGF BB	Basal Media vs. TGFβ1	PDGF BB vs. TGFβ1
**N (cells analyzed)**	79	74	58			
* Cell Area (μm^2^)	517 (318, 721)	1284 (889, 1638)	527 (396, 861)	*p* < 0.01	NS	*p* < 0.01
* Cell Length (μm)	81.6 (25.9, 115.1)	162.5 (126.6, 210.5)	65.5 (37.4, 100.3)	*p* < 0.01	NS	*p* < 0.01
* Length/Breadth	4.4 (1.4, 6.7)	4.9 (2.9, 9.0)	3.1 (1.4, 5.8)	*p* < 0.05	NS	*p* < 0.01

* Non-parametric data are presented as: Median (25th percentile, 75th percentile); *p* values are from ANOVA on Ranks; NS = not statistically significant. For each condition, cells from four different matrices were combined for the analysis.

## Supplementary Materials

The following are available online at https://www.mdpi.com/2306-5354/7/3/89/s1, Figure S1: Differences in cell connectivity and morphology following culture in basal media, PDGF BB and TGFβ1; Figure S2: Comparison of fibrin, fibronectin, and F-actin labeling patterns; Figure S3: Method used for calculating the correlation between actin, fibrin and fibronectin patterning; Table S1: NRK Cell Morphology; Video S1: Time-lapse sequence of LifeAct (green) and fibrin (cyan) patterning by an HTK cell after overnight incubation in PDGF BB, Video S2: Time-lapse sequence of LifeAct (green) and fibrin (cyan) patterning by an HTK cell after overnight incubation in TGFβ1, Video S3: Time-lapse sequence of LifeAct (green in overlay on left), fibrin (cyan in overlay on left) and fibronectin (red in overlay on left, also shown alone in right panel) patterning by a group of HTK cells after 36 h of incubation in PDGF BB, Video S4: 3D rotational sequence of HTK cells fixed after 48 h of incubation with TGFβ1 and labeled for F-actin (red), fibronectin (green) and fibrin (cyan).
