# Supplementary material for: Coupling of Fibrin Reorganization and Fibronectin Patterning by Corneal Fibroblasts in Response to PDGF BB and TGFβ1
Source: Bioengineering (Basel). 2020 Aug 7;7(3):89. doi: 10.3390/bioengineering7030089 (PMC7552779; doi:10.3390/bioengineering7030089)
Supplement: Supplementary file 1 [file bioengineering-07-00089-s001.zip › bioengineering-831352-suppl-final/bioengineering-831352-final supplementary.pdf]

bioengineering

MDPI

Supplementary Materials

Coupling of Fibrin Reorganization and Fibronectin Patterning by Corneal Fibroblasts in Response to PDGF BB and TGFβ1

Miguel Miron-Mendoza, Dalia Vazquez, Nerea García-Rámila, Hikaru R. Ikebe and W. Matthew Petroll *

Department of Ophthalmology, UT Southwestern Medical Center, Dallas, 75390 TX, USA; Miguel.Miron@UTSouthwestern.edu (M.M.-M.); daliavazquez77@outlook.com (D.V.); nereagarciaramila@gmail.com (N.G.-R.); hikaru.ikebe@utsouthwestern.edu (H.R.I.)
* Correspondence: matthew.petroll@utsouthwestern.edu

Received: 26 May 2020; Accepted: 5 August 2020; Published: date

Figure S1: Representative maximum intensity projections of F-actin (green) and nuclei (blue) for HTK cells in 3-D fibrin matrices, following 48 h of culture in basal media (left column), PDGF BB (middle column) or TGFβ1 (right column). Note that cells are more elongated and more interconnected in PDGF BB as compared to basal media or TGFβ1. Scale bar = 75 μm.

Figure S2. Verification that the NH3 ester signal detected is only from fibrin fibers. In this representative image, HTK cells were cultured in a fluorescently labeled 3D fibrin matrix for 48 h in media containing PDGF BB. Subsequently, cells were fixed and labeled for F-actin and fibronectin. (A) Overlay of fibrin (red) and fibronectin (green). Note that in addition to regions with strong colocalization of fibronectin with fibrin (yellow labeling, arrowheads), in many areas fibronectin labeling occurs along the edge of fibrin fibrils (arrows) and the fluorescent signal does not completely overlap. In addition, areas of fibronectin accumulation are observed in areas where no fibrin signal is present. Finally, short segments of fibronectin labeling are often observed along longer fibrin fibers, and there is no increase in the fibrin signal intensity in the segments where fibronectin is present, as would be expected if the fibronectin had been labeled with the NH3 ester. (B) Overlay of fibrin (red) and F-actin (green). Note that in addition to regions with colocalization of F-actin and fibrin (arrowheads), in many areas F-actin labeling is completely uncorrelated with the fibrin fibrils (arrows), including regions along the cell membrane.

Figure S3. Method used for calculating the correlation between actin, fibrin and fibronectin patterning. Changes in pixel intensities were measured across the cell body and between neighboring cells where fibrin compaction was observed. In this example, HTK cells were cultured in a fluorescently labeled 3D fibrin matrix for 48 h in media containing PDGF BB (to stimulate cell spreading). Subsequently, cells were fixed and labeled for F-actin and fibronectin. Images on the left are maximum intensity projections of z-series from each individual channel. Using Image J software, lines were traced across the cell body (red) or between neighboring cells (white), using exactly the same x-y positions for each channel. Subsequently the changes in pixel intensity corresponding to the traced lines for each channel were stored and plotted. Correlation was calculated comparing the linear relationship between actin, fibronectin, and fibrin fibers across the cell body and between neighboring cells. Red and white bars are 40 μm.

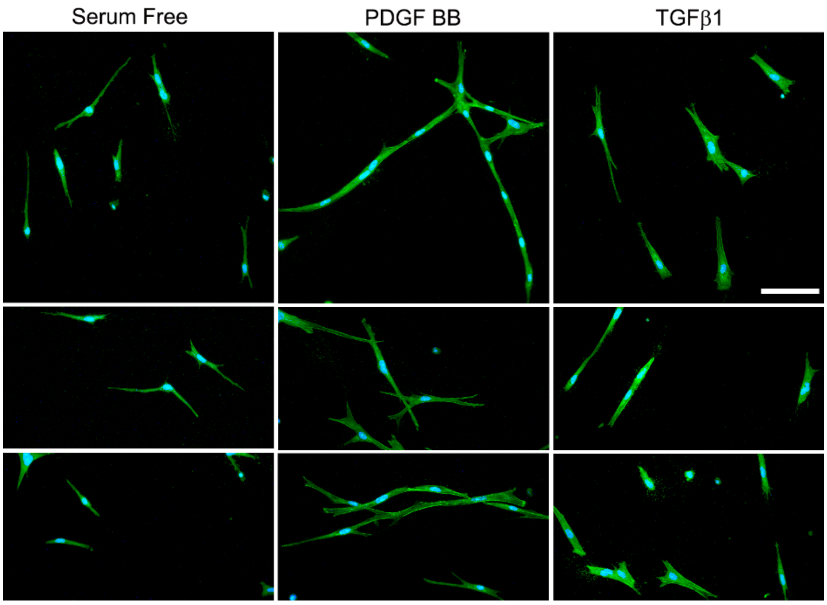

Figure S1. Differences in cell connectivity and morphology following culture in basal media, PDGF BB and TGFβ1

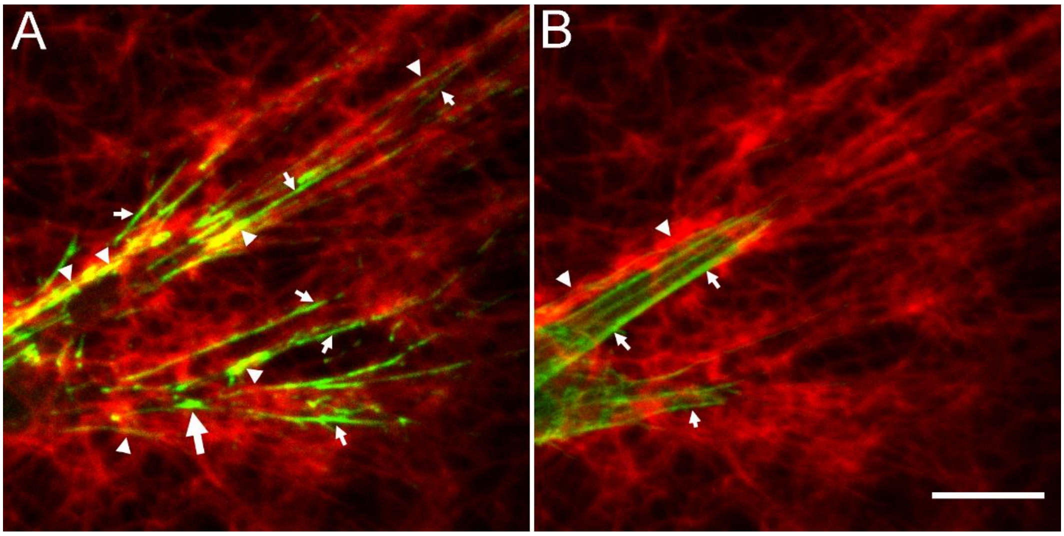

Figure S2. Comparison of fibrin, fibronectin, and F-actin labeling patterns.

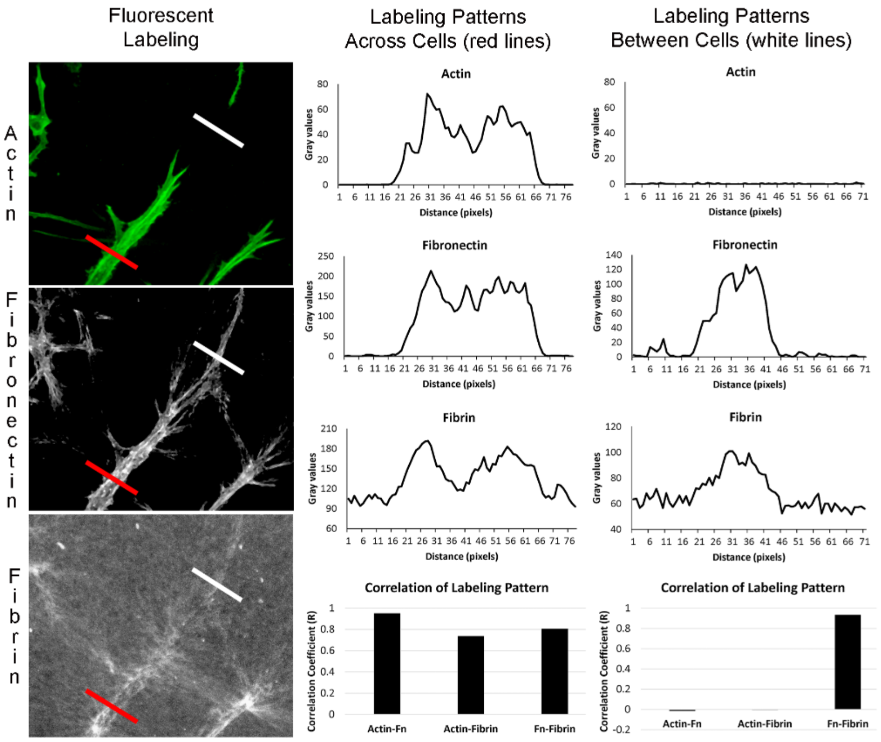

Figure S3. Method used for calculating the correlation between actin, fibrin and fibronectin patterning.

Table S1. NRK Cell Morphology.

	**Basal Media**	**PDGF BB**	**TGFβ1**	**Basal Media vs PDGF BB**	**Basal Media vs TGFβ1**	**PDGF BB vs TGFβ1**
**N (cells)**	28	23	25			
**Cell Area (μm^2)**	737 ± 255	963 ± 390	708 ± 269	P < 0.05	NS	P < 0.05
***Cell Length (μm)**	88.1 (74.2, 105.0)	155.9 (119.2, 235.1)	102.9 (78.1, 150.6)	P < 0.01	NS	P < 0.05
***Length/Breadth**	1.6 (1.3, 2.4)	3.6 (2.1, 5.3)	1.9 (1.4, 2.8)	P < 0.05	NS	P < 0.01

*Non-parametric data are presented as: Median (25th percentile, 75th percentile);

P values are from ANOVA (Cell Area) or ANOVA on Ranks (Cell length, Length/Breadth); NS = not significant

For each condition, cells from two different matrices were combined for the analysis

© 2020 by the authors. Submitted for possible open access publication under the terms and conditions of the Creative Commons Attribution (CC BY) license (http://creativecommons.org/licenses/by/4.0/).
