# Supplementary material for: Coupling of Fibrin Reorganization and Fibronectin Patterning by Corneal Fibroblasts in Response to PDGF BB and TGFβ1
Source: Bioengineering (Basel). 2020 Aug 7;7(3):89. doi: 10.3390/bioengineering7030089 (PMC7552779; doi:10.3390/bioengineering7030089)
Supplement: Supplementary file 1 [file bioengineering-07-00089-s001.zip › bioengineering-831352-suppl-final/SupplementalMaterial/Table S1 new.docx]

| **Table S1: NRK Cell Morphology** | | | | | | |
| --- | --- | --- | --- | --- | --- | --- |
|  | **Basal Media** | **PDGF BB** | **TGFβ1** | **Basal Media vs PDGF BB** | **Basal Media vs TGFβ1** | **PDGF BB vs TGFβ1** |
| **N (cells)** | 28 | 23 | 25 |  |  |  |
| **Cell Area (μm^2^)** | 737 + 255 | 963 + 390 | 708 + 269 | P < 0.05 | NS | P < 0.05 |
| ***Cell Length (μm)** | 88.1 (74.2, 105.0) | 155.9 (119.2, 235.1) | 102.9 (78.1, 150.6) | P < 0.01 | NS | P < 0.05 |
| ***Length/Breadth** | 1.6 (1.3, 2.4) | 3.6 (2.1, 5.3) | 1.9 (1.4, 2.8) | P < 0.05 | NS | P < 0.01 |
| *Non-parametric data are presented as: Median (25th percentile, 75th percentile); | | | | | | |
| P values are from ANOVA (Cell Area) or ANOVA on Ranks (Cell length, Length/Breadth); NS = not significant | | | | | | |
| For each condition, cells from two different matrices were combined for the analysis | | | | | | |
