# Supplementary figures and images for: Coupling of Fibrin Reorganization and Fibronectin Patterning by Corneal Fibroblasts in Response to PDGF BB and TGFβ1
Source: Bioengineering (Basel). 2020 Aug 7;7(3):89. doi: 10.3390/bioengineering7030089 (PMC7552779; doi:10.3390/bioengineering7030089)

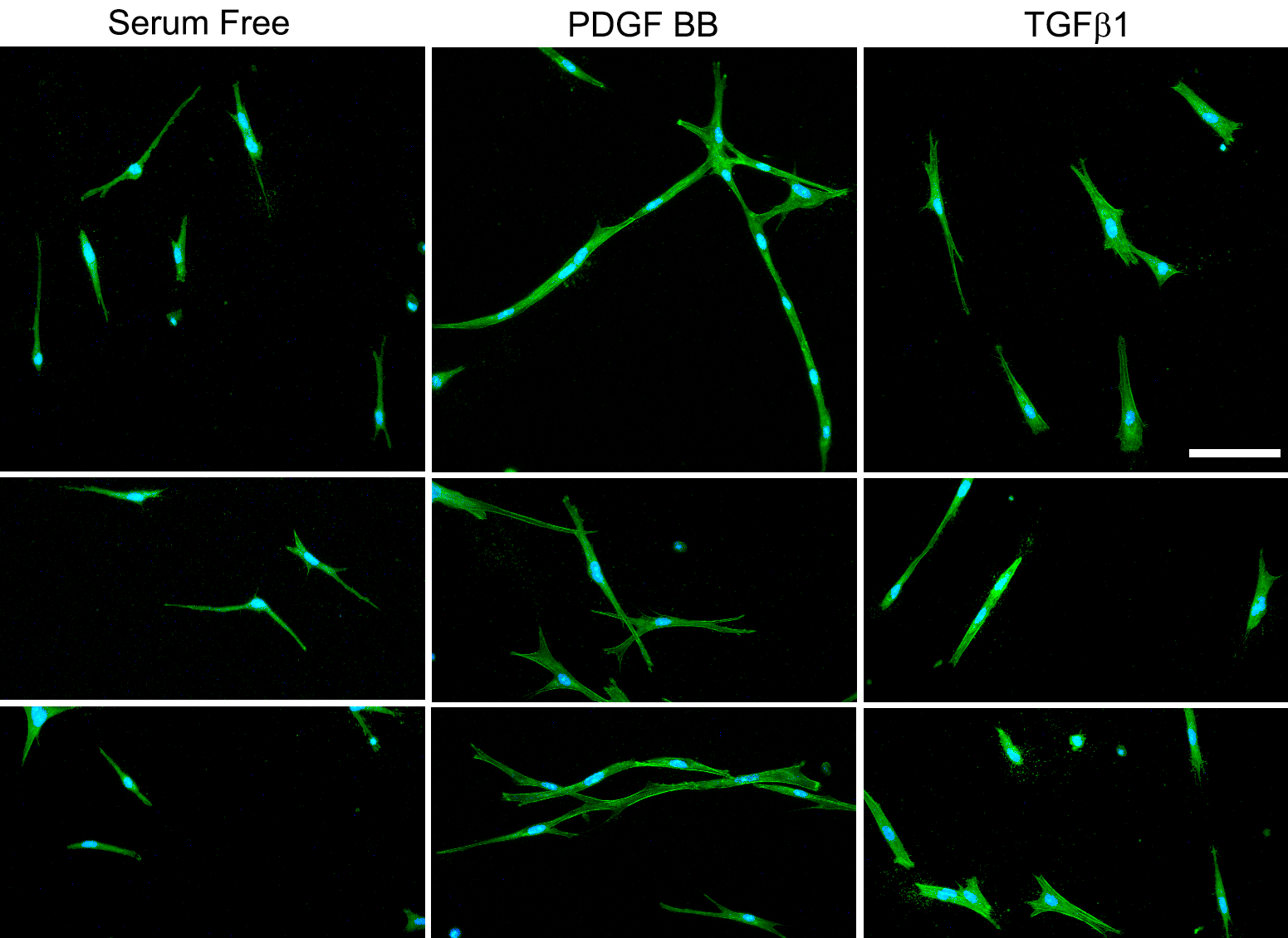

Supplement: Supplementary file 1 [file bioengineering-07-00089-s001.zip › bioengineering-831352-suppl-final/SupplementalMaterial/SupplementalFigure1_Final.tif]

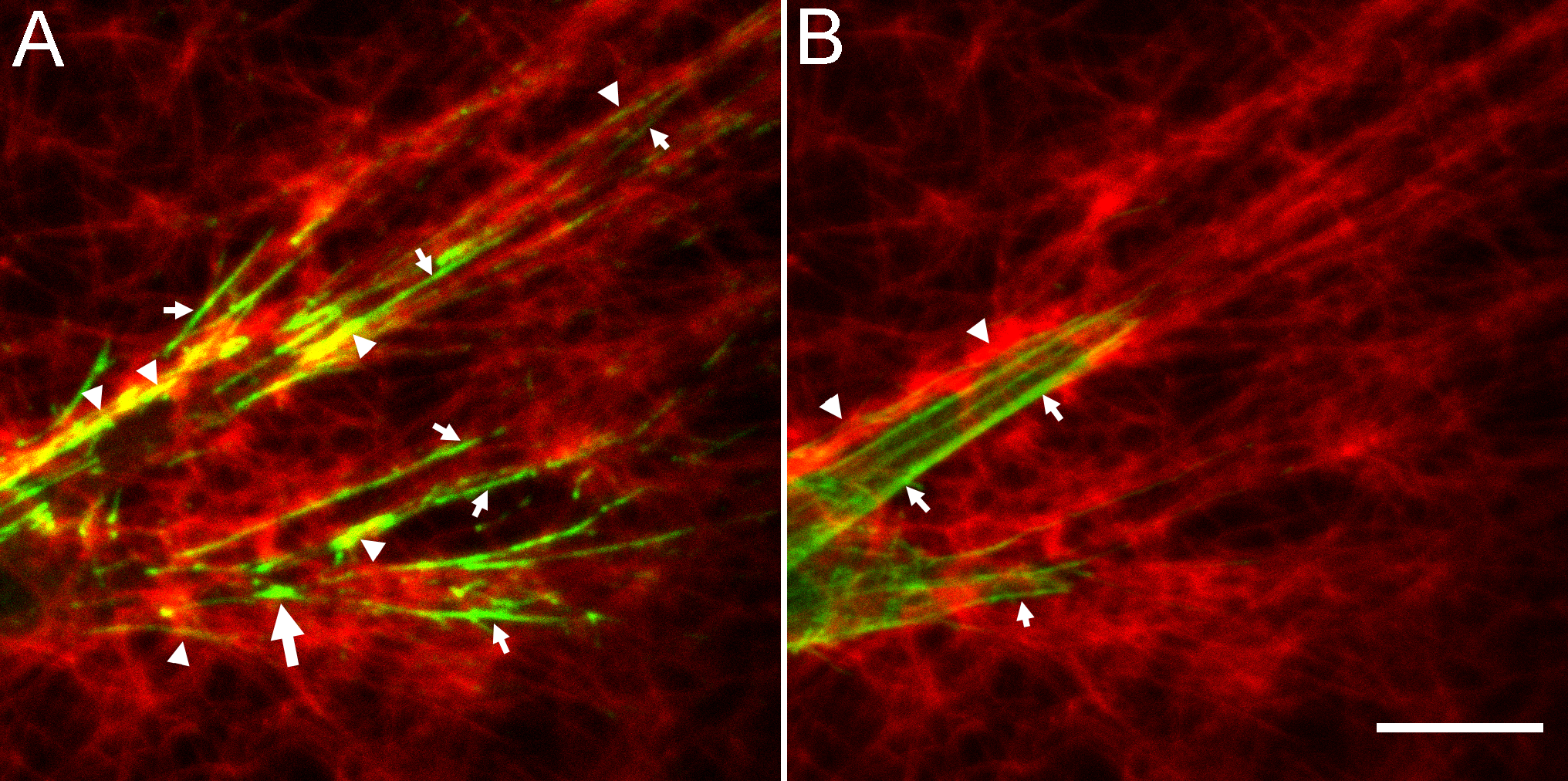

Supplement: Supplementary file 1 [file bioengineering-07-00089-s001.zip › bioengineering-831352-suppl-final/SupplementalMaterial/SupplementalFigure2_Final.tif]

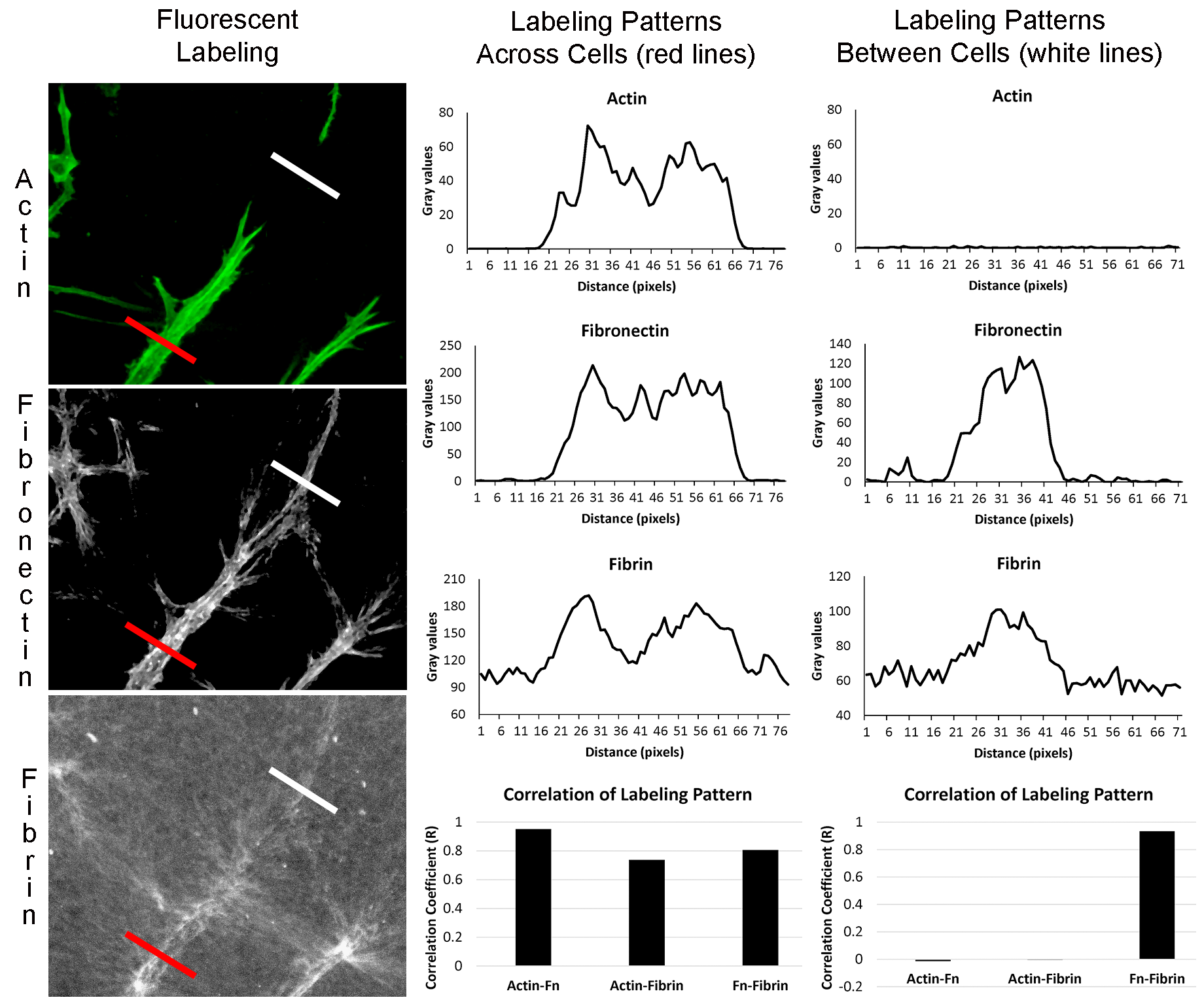

Supplement: Supplementary file 1 [file bioengineering-07-00089-s001.zip › bioengineering-831352-suppl-final/SupplementalMaterial/SupplementalFigure3_Final.tif]
